# Impact of COVID-19 on the Belgian HIV epidemic: slowdown of HIV transmission and testing and adaptation of care

**DOI:** 10.1186/s12879-022-07879-1

**Published:** 2022-12-03

**Authors:** Dominique Van Beckhoven, Ben Serrien, Marion Montourcy, Chris Verhofstede, Dorien Van den Bossche, Agnes Libois, Deborah De Geyter, Thierry Martin, Sandra Van den Eynde, Bea Vuylsteke, Gilles Darcis, Karlijn van Halem, Eric Florence, Jessika Deblonde, Belgian Research on AIDS and HIV Consortium (BREACH), Nathalie Ausselet, Marie-Luce Delforge, Rémy Demeester, Paul De Munter, Jean-Christophe Goffard, Benoït Kabamba, Rembert Mertens, Peter Messiaen, Michel Moutschen, Denis Pierard, Dolorès Vaira, Linos Vandekerckhove, Sigi Van den Wijngaert, Kristel Van Laethem, Jens Van Praet, Jean-Cyr Yombi

**Affiliations:** 1 Department of Epidemiology and Public Health, Sciensano, Brussels, Belgium; 2 Faculty of Public Health, Université Catholique de Louvain, Brussels, Belgium; 3 Department of Diagnostic Sciences, Aids Reference Laboratory, Ghent University, Ghent, Belgium; 4 Department of Clinical Sciences, Institute of Tropical Medicine, Antwerp, Belgium; 5 Department of Infectious Diseases, Saint-Pierre University Hospital, Université Libre de Bruxelles, Brussels, Belgium; 6 Department of Clinical Biology, Laboratory of Microbiology, Universitair Ziekenhuis Brussel, Vrije Universiteit Brussel, Brussels, Belgium; 7 Plateforme Prévention SIDA, Brussels, Belgium; 8 Sensoa, Antwerp, Belgium; 9 Department of Public Health, Institute of Tropical Medicine, Antwerp, Belgium; 10 Department of Internal Medicine and Infectious Diseases, Liège University Hospital, Liège, Belgium; 11 Department of Infectious Diseases and Immunity, Jessa Hospital, Hasselt, Belgium

**Keywords:** HIV trend, COVID-19 impact, HIV diagnosis, HIV testing, HIV care

## Abstract

**Background:**

To gain insight into the impact of the COVID-19 pandemic and containment measures on the HIV epidemic and services, this study aims to describe HIV trends in 2020 and compare them with previous years.

**Methods:**

Belgian national HIV surveillance data 2017–2020 were analysed for trends in HIV testing, HIV diagnoses, VL measurements, ART uptake and PrEP purchase. Descriptive statistics from 2020 are compared to annual averages from 2017 to 2019 (proportional difference, %).

**Results:**

In 2020, 725 HIV infections were diagnosed in Belgium (− 21.5% compared to 2019). The decline was most pronounced during the first lockdown in April–May but also present in July–December. The number of HIV tests performed decreased by 17.6% in 2020, particularly in March–May and October–December (− 57.5% in April and -25.4% in November 2020 compared to monthly 2017–19 numbers). Diagnosis of acute HIV infections decreased by 47.1% in 2020 (n = 27) compared to 2019 (n = 51). Late HIV diagnoses decreased by 24.7% (95% CI [− 40.7%; -9.7%]) in 2020 compared to 2019. Of patients in care in 2019, 11.8% interrupted HIV care in 2020 compared to 9.1% yearly in the 3 previous years. The number of HIV patients with VL monitoring per month dropped in March–May 2020, whilst proportions of VL suppression and ART coverage remained above 86% and 98.5% respectively in 2020. PrEP purchases, number of purchasers and starters dropped during April–May 2020 (respectively − 45.7%, − 47.4%, − 77.9% in April compared to February 2020).

**Conclusions:**

The significant decrease in HIV diagnoses in Belgium in 2020 coincided with the COVID-19 pandemic and following containment measures, particularly in April–May during the first lockdown. A slowdown of HIV transmission due to reduced HIV risk exposure is suggested by the halving in diagnosis of acute HIV infections in March-December 2020 compared to the previous year, and the adaptive decrease in PrEP use and PrEP initiation from April onwards. Despite a slight increase in HIV care interruptions, the indicators of quality of HIV care remained stable. Access to prevention, testing and care for all people living with HIV and at risk of acquiring HIV is a priority during and after times of pandemic.

## Background

The COVID-19 pandemic has created an unprecedented public health emergency worldwide. In response to multiple outbreaks from January 2020 onwards, global measures were put in place to contain the circulation of SARS-CoV-2. In Belgium, restrictions culminated during two lock-down periods in 2020: from the 18th of March with progressive reopening from the 4th of May, and from the 2nd of November with gradual release from the 1st of December. Travel restrictions, ban on public gatherings and the closure of schools and non-essential services were imposed, as well as the suspension of non-urgent medical care and prevention services.

Several observational studies have shown the major impact of the COVID-19 pandemic and the resulting measures on the provision of HIV services as well as on the evolution of the HIV epidemic [1–3]. Health promotion, prevention and support organisations tried to adapt their services to this exceptional situation of reduced physical contact and lock-down. In consequence, ongoing programs were interrupted or reprogrammed to focus on the urgent needs related to COVID-19 prevention and care [4]. Interruption of medical services had an impact on the provision of healthcare for people with HIV (PWH) and pre-exposure prophylaxis (PrEP). Moreover, health facilities and staff, particularly infectious disease specialists, were mobilized to contribute to the pandemic response. As a consequence, non-urgent HIV-related medical care was cancelled or converted to telephone or online consultations [5].

The objective of this study is to describe the trends of the HIV epidemic in the first year of the COVID-19 pandemic in Belgium and compare them with previous years.

## Methods

### Study setting and population

In Belgium, all HIV diagnoses and viral load measurements are performed by specialized AIDS Reference Laboratories (ARL). The HIV Reference Centers (HRC) are specialized tertiary care structures offering multi-disciplinary care for PWH. More than eighty percent of HIV patients are followed up in those centers. Alternatively, HIV patients are followed up in other hospitals and, although less frequently, in primary care.

PrEP delivery has been implemented and reimbursed in Belgium since June 2017. Emtricitabine/tenofovir as PrEP, in boxes of 30 or 90 pills, might be purchased from public pharmacies, and less frequently from hospital pharmacies (13% of purchases).

Access to HIV testing and care is covered by the national compulsory health insurance for Belgians and non-Belgians legally residing in Belgium. European Union (EU) nationals get similar access through their home country’s health insurance. For undocumented non-EU migrants, access to healthcare is ensured by a process of “Urgent medical aid”, although in practice administrative barriers and restrictions are often faced.

### Data collection

As national HIV surveillance team from Sciensano, the Belgian institute for health, we collect individual HIV diagnostic and viral load data annually from the ARL, including demographic information while the HRC report routine individual demographic, medical, treatment and laboratory data from the patients in care. In addition, claim data on testing for HIV and on PrEP delivery in public pharmacies are obtained from the National Institute for Health and Disability Insurance. This allows for a national exhaustive surveillance covering all people tested for HIV, all those diagnosed with HIV, all those in HIV care and all people using PrEP (except those purchasing PrEP solely in hospital pharmacies).

Sciensano is legally entitled to conduct this HIV surveillance, which has been approved by an independent administrative authority protecting privacy and personal data [6].

### Statistical analysis

We selected surveillance data from the 1st of January 2017 to the 31st of December 2020. Yearly and monthly trends in HIV tests, HIV diagnoses, viral load measurements, ART uptake and PrEP purchase were analysed. Missing data values were enriched through linkages between diagnosis and care registers. Additionally, multiple imputation was applied on the diagnosis data for remaining missing variables, as advised as an adjustment method to account for missing data in HIV surveillance in Europe [7]. This allowed monitoring of the trends in number of HIV diagnoses per demographic group without introducing a bias related to varying missing data over time. The R package “mice” was used to construct 10 multiply imputed datasets [8]. A non-parametric classification and regression tree was used as imputation method for all variables as this is known to preserve interactions between variables and is thus well suited to capture differential changes over time of different groups [9]. Descriptive statistics based on imputed data were pooled across multiply imputed datasets using Rubin’s rules [8] and presented as point estimates and 95% CI. Statistics related to the number of diagnoses, diagnostic tests and PrEP purchases are presented as point estimates as they are based on exhaustive and complete data.

## Results

In 2020, 725 diagnoses of HIV infection were confirmed in Belgium, this is a decrease of 21.5% compared to 2019 (N = 926). In the 3 preceding years (2017–2019), no decline in the number of HIV diagnoses was recorded (mean yearly change = − 0.006%). The decline in the number of HIV diagnoses in 2020 compared to 2017–2019 was particularly pronounced in women (− 25.9%), people < 30 years of age (− 25.6%) and people of Belgian (− 34.6%) and Sub-Saharan African nationalities (-31.3%). Socio-demographic characteristics of diagnosed PWH are presented in Table 1.

**Table 1:** Sociodemographic characteristics of HIV diagnosed people in 2020 and 2017–2019

	Original data		Imputed data^$^		
	2017–2019	2020	2017–2019	2020	Difference (%)**
	N (%)*	N (%)	N (%)*	N (%)	Δ [95% CI]
Sex					
Male	611 (67%)	508 (70%)	617 (68%)	510 (70%)	− 17.3% [− 22.6%; − 11.9%]
Female	286 (32%)	213 (29%)	289 (32%)	215 (30%)	− 25.9% [− 36.8%; − 14.9%]
Missing	9 (1%)	4 (1%)			
Age group					
< 30	234 (26%)	170 (23%)	234 (26%)	174 (24%)	− 25.6% [− 38.3%; − 13.0%]
30–49	489 (54%)	409 (56%)	489 (54%)	409 (56%)	− 16.4% [− 23.8%; − 9.1%]
≥ 50	183 (20%)	142 (20%)	183 (20%)	142 (20%)	− 22.4% [− 37.6%; − 7.2%]
Missing	0 (0%)	4 (1%)			
Nationality					
Belgian	302 (33%)	182 (25%)	355 (39%)	232 (32%)	− 34.6% [− 43.8%; − 25.5%]
European	126 (14%)	142 (20%)	144 (16%)	159 (22%)	+ 10.8% [− 13.0%; + 34.5%]
Subs. African	218 (24%)	149 (21%)	269 (30%)	185 (26%)	− 31.3% [− 42.8%; − 19.7%]
Other	123 (14%)	131 (18%)	138 (15%)	149 (20%)	+ 7.7% [− 17.3%; + 32.8%]
Missing	137 (15%)	121 (16%)			
Mode of infection					
MSM	344 (38%)	248 (34%)	413 (46%)	336 (46%)	− 18.5% [− 27.9%; − 9.2%]
Heterosexual	345 (38%)	247 (34%)	452 (50%)	353 (49%)	− 21.9% [− 3.05%; − 13.3%]
PWID	12 (1%)	6 (1%)	18 (2%)	13 (2%)	− 26.4% [− 94.5%; + 41.8%]
Other	14 (2%)	15 (2%)	23 (3%)	23 (3%)	− 1.7% [− 65.4%; − 62.0%]
Missing	191 (21%)	209 (29%)			

*Yearly average of N (%)

**Proportional difference in number of diagnoses in 2020 compared to the mean of the 3 preceding years: $\Delta =100\mathrm{\% }\times \frac{{N}_{2020}-{\underline{N}}_{17-19}}{{\underline{N}}_{17-19}}$

^$^The descriptives for the imputed data show the point estimates of N (%) (rounded average of the counts and percentages across the 10 imputed datasets). For the change score Δ we show both the point estimate (average across imputed datasets) and the variability (95% CI)

**Fig. 1:**
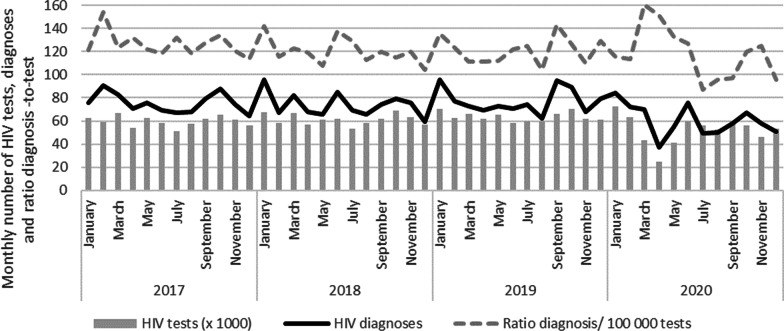
Monthly number of HIV diagnoses, HIV tests and ratio diagnoses per 100,000 tests, 2017–2020, Belgium

**Fig. 2:**
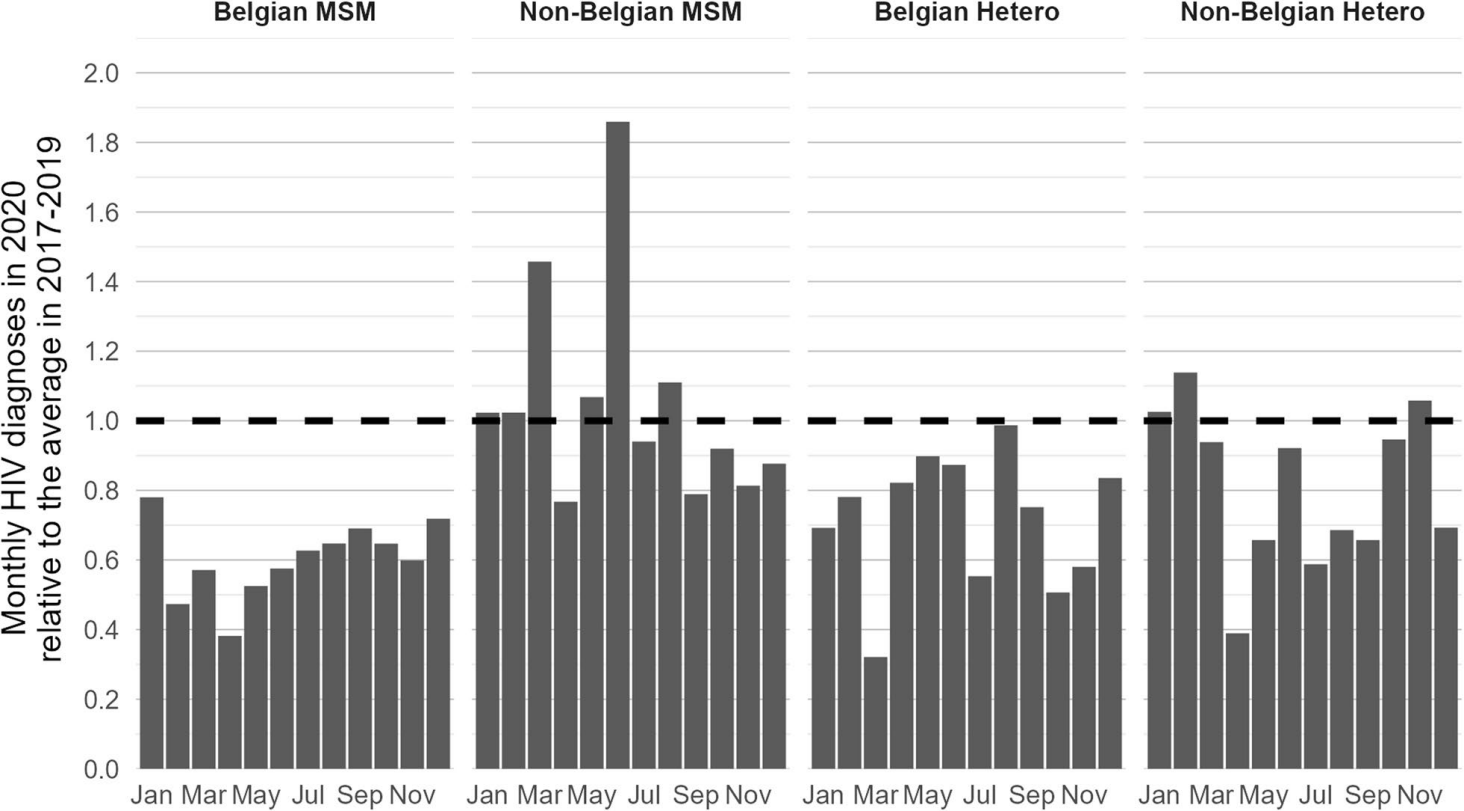
Monthly number of HIV diagnoses by nationality and mode of transmission in 2020, expressed as a ratio relative to the mean number of diagnoses in 2017–2019

The decline in HIV diagnoses in 2020 was most pronounced during the first lockdown from April to May but was also noticed from July to the end of 2020 (Fig. 1). A clear monthly reduction of the number of HIV diagnoses was observed among Belgian MSM and heterosexuals throughout 2020 (Fig. 2). For non-Belgians, a higher variability per month was seen with a pronounced drop in April.

A total of 629 063 HIV tests were performed in 2020, representing a testing rate of 56 per 1000 population. Between 2019 and 2020, the number of tests performed decreased by 17.6%. The number of HIV tests carried out from March to May and from October to December 2020 decreased substantially compared to the monthly number of tests carried out in 2017–19 (− 57.5% in April and − 25.4% in November 2020). From March to May 2020, the ratio of new diagnoses per test was higher (1.5 new diagnoses/1000 tests) than the annual average of previous years of 1.2 new diagnoses/1000 tests. It reduced in the following months and was very low from July to September 2020 (0.9 new diagnoses/1000 tests) (Fig. 1).

The number of laboratory-identified acute infections, defined by the presence of p-24 antigen or plasma viral RNA in combination with a negative or indeterminate immunoassay result (InnoLIA or Geenius), decreased by 47.1% in 2020 compared to 2019 (n = 27 and n = 51 respectively). No acute infections were diagnosed in April and November 2020, and the number remained low (1–2 per month) in the following months until the end of the year, except for September (4 acute infections). Belgian MSM have the highest probability of being diagnosed during acute infection (54.5% of the acute infections between 2017 and 2020 were Belgian MSM), and it is among them that the largest decrease was observed (-67.5% in 2020 compared to 2019; 95% CI [− 89.4%; − 45.6%]).

In 2020, 34.2% of the infections were diagnosed late (defined as CD4 < 350 cells/mm3 or AIDS and no indication of a recent infection). The proportion of late diagnoses was higher among non-Belgians (37.1%) than among Belgians (27.8%). Overall, the number of late HIV diagnoses decreased by 24.7% (95% CI [− 40.7%; − 9.7%]) in 2020 compared to 2019. The decrease was most pronounced between March and May and between July and August, while the number of late diagnoses in the rest of the year was similar to previous years. The number of late diagnoses was reduced by 28.3% (95% CI [− 50.2%; − 6.4%]) among MSM, by 17.4% (95% CI [− 32.3%; − 2.4%]) among heterosexuals, by 40.6% (95% CI [− 57.8%; − 23.4%]) among Belgians and by 10.3% (95% CI [− 26.5%; + 5.8%]) among non-Belgians.

In 2020, 17,018 HIV patients were in care in Belgium. While the number of patients in HIV care increased steadily by a mean of 492 persons each year from 2017 to 2019, it reduced by 139 in 2020. Of the 17,157 patients in care in the preceding year, 2032 (11.8%) did not have a record of CD4 measurement, VL measurement or HRC medical visit in 2020. In comparison, in 2017–2019, an average of 9.1% was interrupting HIV care each year.

**Fig. 3:**
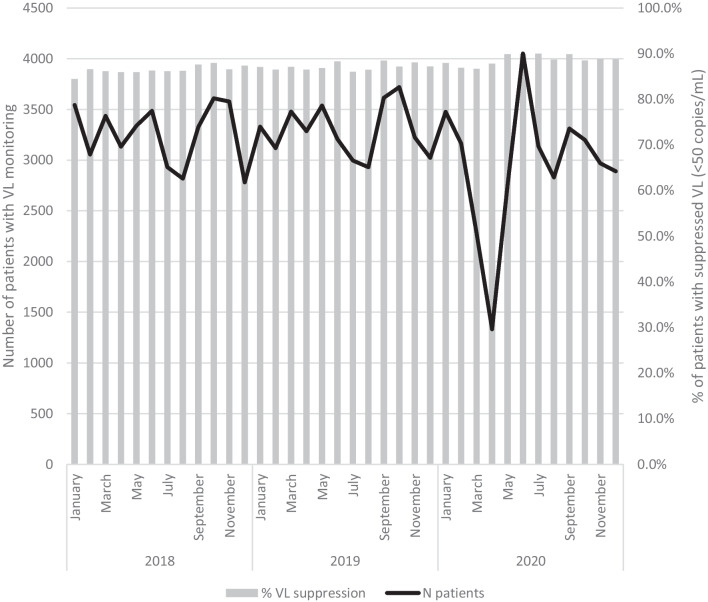
Number of HIV patients with viral load monitoring (at least one viral load measured during the month) and proportion of patients with suppressed VL (< 50 copies/mL) by month, 2018–2020, Belgium

The monthly number of HIV patients with viral load monitoring decreased significantly during the first COVID-19 lockdown from March to May 2020 (Fig. 3). A slight catch-up was then observed in June. From July onwards, the number of patients undergoing virological monitoring was comparable to previous years. Throughout 2020, the proportion of VL suppression among monitored patients remained above 86%. Data on antiretroviral therapy (ART) in the HRC showed that ART coverage remained high among patients in HIV care at 98.5% in 2020.

**Fig. 4:**
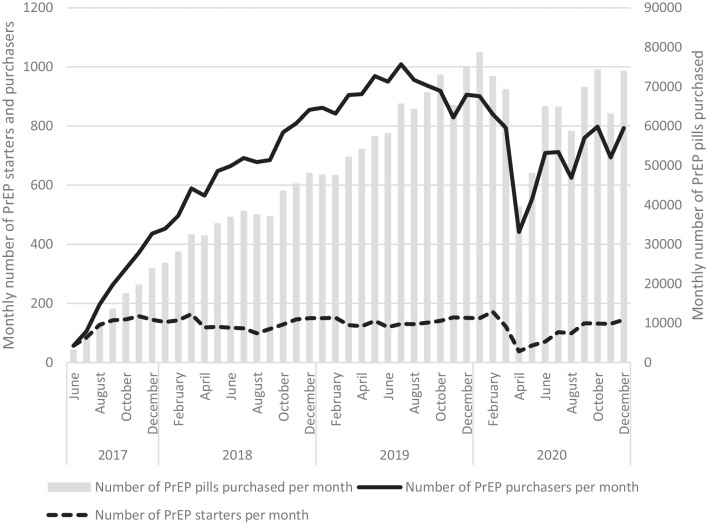
Monthly number of PrEP starters, purchasers and number of PrEP pills purchased from public pharmacies, 2017–20, Belgium

Since June 2017, the number of PrEP users has steadily increased. In 2020, 3983 persons purchased PrEP in public pharmacies, which represents an increase of 12.1% compared to 2019. The overall number of PrEP pills purchased in 2020 remained stable compared to 2019 (respectively 776,790 vs 727,770). PrEP purchases and number of purchasers dropped during the months of April and May 2020 (by respectively 33,180 (− 45.7%) and 398 (− 47.4%) in April compared to February 2020) (Fig. 4). Number of PrEP starters also reduced during the lock-down by 134 (− 77.9%) in April compared to February 2020.

## Discussion

Our study provided strong indications that the decrease in HIV diagnoses in Belgium in 2020 was linked to the COVID-19 pandemic and the imposed containment measures. Indeed, the decline of HIV diagnoses was most pronounced during the first lockdown in April–May. In the following months, with the exception of June, the number of diagnoses remained significantly lower than in previous years. In contrast, HIV care indicators such as viral suppression and ART coverage remained good though a slight increase in HIV care interruption was observed in 2020.

As also observed in other countries, the decrease in HIV diagnoses in Belgium largely coincided with a reduction in testing activities [1, 10]. This reduction was reported in health care facilities and in community based testing services which target specific key populations [4]. The amount of tests performed depends on the available and accessible offer and the clients’ or patients’ demand, which in turn is also influenced by (self-perceived) risk of HIV infection.

During the months of March to May 2020, whilst access to testing facilities was very limited leading to a decrease in test offer, the ratio of diagnoses per test was higher than the annual average of previous years. This suggests a restriction of testing towards those at high risk or with suggestive clinical symptoms. Between June and September, access to testing normalised with a similar number of tests per month as in previous years. The ratio of diagnoses per test was very low in the summer months, suggesting an extension of testing to less urgent or less specific indications, which were postponed during the lock-down period.

Several studies suggest that HIV transmission may have been reduced by the decrease in sexual interactions with casual partners due to the restriction of physical and social contacts during the periods of strict confinement [11]. An online survey among MSM in Belgium showed a sharp drop in the number of casual sex partners during the first lock-down period from March to May 2020 [12]. Similar results were observed in studies in the Netherlands [13], United Kingdom [14, 15] and Australia [16].

A first observation that is in line with those studies showing a lower risk of HIV transmission in this period is the halving of the number of acute HIV diagnoses from March 2020 to the end of the year compared to the previous year. Diagnoses during the acute phase of HIV infection have decreased significantly more than the HIV diagnoses in general, while during acute infection a large majority of patients present with clinical symptoms, which may draw clinicians’ to this differential diagnosis, as opposed to established HIV infection. However, it cannot be excluded that some diagnoses of acute infections may have been missed as HIV acute infection may mimic COVID-19 symptoms [17].

A second observation in the same direction is the decrease in the purchase of PrEP pills and the number of PrEP purchasers from April 2020 onwards, meaning that some users have reduced or suspended their PrEP use, and very few initiated PrEP. Studies among PrEP users indicated that they tailor their PrEP use according to their risk exposure [18–20]. Continued use of PrEP during periods of lockdown was found to be associated with having sex with casual partners [12]. It can therefore be assumed that PrEP use patterns were adapted by users according to the perceived needs, potentially allowing PrEP to continue playing its role in reinforcing combination prevention.

HIV diagnoses among heterosexuals are still mainly reported in people of sub-Saharan African origin followed by people of other non-Belgian nationalities. Among non-Belgians, the decrease in HIV diagnoses was mainly observed during the COVID-19 lockdown periods when travel restrictions were in place. The pandemic has had a major impact on international migration flows to Belgium (-15.5% international immigrations in 2020 compared to 2017–19 [21]) and in consequence, most likely, on the number of non-Belgian PWH whose diagnosis was confirmed after arrival in Belgium.

In parallel to the decrease in the overall number of HIV diagnoses, there was a decrease in the number of late diagnoses. On the one hand, travel restrictions and reduced migration during the lockdown periods may have had an impact on the number of late diagnoses among non-Belgians, although this number decreased only minimally compared to previous years. On the other hand, limited accessibility to testing facilities also played a role and may have increased the delay in diagnosis for some people, as reported in the Netherlands where more advanced disease was observed at entry in clinical care after the lockdown periods [22]. Though no rebound with an increase in late diagnoses post lock-down was noticed in Belgium until end 2020, which is a preliminary reassuring signal. The trend in late diagnoses in the following year will be monitored to reappraise the extent to which HIV diagnoses were missed in 2020.

As a result of the social and physical distancing, as well as shift from the infectious diseases dedicated staff to COVID-19 care, the organization of the HIV-care had to be adapted during the periods of lockdown, with an impetus for alternative strategies such as telemedicine [5, 23]. This adaptation of care is illustrated by the reduced number of viral load tests performed during the months of March to May, linked to the postponement of these tests in clinically stable patients. A similar trend was observed in a monocentric study in Belgium [23]. Nevertheless, the antiretroviral treatment coverage remained high in 2020 and very effective in controlling the viral load of HIV-positive patients in care. The feasibility and positive experience of this digital switch supports its use in future routine HIV care.

Similarly, prevention services had to be adapted during lock-down: renewal of the PrEP prescriptions could be done remotely by the physician allowing the collection of the PrEP pills at the pharmacy. Field prevention and support organizations reinvented their action package to adapt to the pandemic by informing PWH about COVID-19 vaccines and providing support in making vaccine appointments. The link with the community was maintained by switching to online prevention, support and sharing groups and supportive phone calls. Testing was supported through the distribution of HIV self-tests (T. Martin, Director Plateforme Prévention SIDA, personal communication, April 19, 2022). Harm-reduction services also had to adapt their service offer during the lock-down periods by limiting or stopping the public reception, introducing online support, adjusting syringe exchange programs and providing information and education on COVID-19 prevention [24].

Finally, structural inequalities related to poverty and migration may have been exacerbated during the COVID-19 pandemic, potentially limiting access to health services for vulnerable populations even more than before [25–27]. Our results show indeed a slight increase in HIV care interruption in 2020. The results of a study conducted among PWH revealed that the negative impact of COVID-19 was more important in the most vulnerable PWH. The reported reasons include an increased fear of COVID-19 infection, greater sense of isolation for PWH living alone, deterioration of financial situation especially for those in the informal economy, and increased uncertainty of legal status for asylum seekers. For some, this led to weight gain and bad habits such as alcohol use and other addictions (Thierry Martin, Director Plateforme Prévention SIDA, personal communication, April 19, 2022). Therefore it is essential, particularly during a pandemic period, that prevention and testing facilities as well as care are inclusive and accessible to all affected populations.

## Conclusions

The pandemic and the containment measures affected several determinants of HIV infection, diagnosis and care as discussed above including access to prevention, testing and care services, sexual behaviour and migration dynamics. The COVID-19 pandemic significantly slowed down HIV transmission in 2020, mainly during the lockdown periods but also in the following months. Missed HIV diagnoses related to testing restrictions might have occurred but this will only be confirmed by looking at future trends. The resilient and adaptive health care system has made it possible to maintain the quality of HIV care in Belgium, although the number of HIV care interruptions increased slightly. After this long-lasting crisis, health policies should aim to support health professionals and organizations in resuming routine care and support for all PWH particularly for the most vulnerable which are at greater risk of loss to follow-up. Prevention and testing services should remain accessible during times of lockdown with an increased focus on those who continue to engage in high risk sexual practices during the pandemic.

## Data Availability

The data that support the findings of this study are available on request from the corresponding author [DVB] upon approval of a project proposal to the HIV surveillance Steering Committee and clearance by the Belgian Information Security Committee.

## Abbreviations

ART: Antiretroviral therapy
HIV: Human Immunodeficiency virus
HRC: HIV reference center
MSM: Men having sex with men
PrEP: Pre-exposure prophylaxis
PWH: People with HIV
